# Development and validation of an ultrasound-based AI-radiomics model for diagnosing and risk-stratifying gastrointestional stromal tumors: a retrospective diagnostic study

**DOI:** 10.1186/s12880-025-02050-z

**Published:** 2025-11-27

**Authors:** Yang Liu, Hongzhang Wang, Liang Deng, Xuemei He

**Affiliations:** 1 Department of Ultrasound, The First Affiliated Hospital of Chongqing Medical University, No. 1 Youyi Road, Yuzhong District, Chongqing, 400016 China; 2 School of Resources and Safety Engineering, Chongqing University, No. 174 Shazhengjie, Shapingba District, Chongqing, 400044 China; 3 Department of Gastroenterology, The First Affiliated Hospital of Chongqing Medical University, No. 1 Youyi Road, Yuzhong District, Chongqing, 400016 China

**Keywords:** Gastrointestinal stromal tumors, Artificial intelligence, Radiomics, Endoscopic ultrasonography

## Abstract

**Background/Aims:**

Accurate discrimination between gastrointestinal stromal tumors (GISTs) and leiomyomas is essential for guiding clinical management. This study developed and validated three predictive models: a baseline characteristics model (BCM), an endoscopic ultrasound-based morphological model (EUS-Morph), and a multimodal fusion model (MMF) incorporating clinical, EUS, and radiomics features. Their diagnostic performance in distinguishing GISTs from leiomyomas was systematically evaluated, along with their potential utility for GIST risk stratification.

**Materials and methods:**

A retrospective analysis was conducted on 3,393 EUS images from 265 pathologically confirmed GIST/leiomyoma patients to differentiate tumors and stratify GIST risk. The diagnostic performance of various models was compared with each other and against endoscopists in the development cohort.

**Results:**

We screened out several key independent variables that distinguish leiomyoma from GIST, including the Length/Short-axis (L/S) Ratio. After comparison, The MMF Model demonstrated optimal diagnostic and stratification performance, achieving the areas under the curve (AUC) values of 0.975 and 0.992, respectively. In contrast, the AUC range for diagnosing GIST by three experienced endoscopists was 0.68–0.70, and the AUC range for risk stratification was 0.72–0.74. Its performance in diagnosing GIST is over 40% higher than that of experienced endoscopists.

**Conclusion:**

All three developed AI models demonstrated favorable performance. Notably, the MMF model, which integrates patient baseline data, clinical characteristics, two-dimensional EUS images and radiomic features, effectively differentiated GISTs from leiomyomas GILs and achieved accurate risk stratification of GISTs. This EUS image-based integrated AI-radiomics model demonstrated improved diagnostic performance compared to endoscopists and may serve as a complementary tool in clinical practice.

**Supplementary Information:**

The online version contains supplementary material available at 10.1186/s12880-025-02050-z.

## Introduction

As the two most frequently encountered subepithelial lesions (SELs), Gastrointestinal stromal tumors (GISTs) and leiomyomas demand distinct therapeutic approaches GISTs often require surgical resection or tyrosine kinase inhibitors, whereas leiomyomas are benign and typically cured by elective excision [1–4]. According to guidelines from the National Comprehensive Cancer Network (NCCN) and the European Society of Gastrointestinal Endoscopy (ESGE), endoscopic ultrasound (EUS) persists as the optimal imaging modality for the characterization of SELs. However, when used alone, EUS has limited capability to accurately differentiate tumor types, and its diagnostic performance heavily relies on the endoscopist’s expertise and experience [5, 6]. Conversely, histopathological confirmation through biopsy or surgical resection carries inherent risks of hemorrhage and potential tumor dissemination [7, 8]. Moreover, GIST prognosis is strongly associated with risk stratification, where high-risk tumors demonstrate significant metastatic potential (35–50% recurrence rate) versus the >90% 5-year survival of low-risk cases [9, 10]. Accurate risk stratification enables optimal prognostic evaluation and guides individualized treatment strategies including surveillance intervals thereby improving long term survival while reducing recurrence rates. However, the current NIH and AFIP criteria require invasive tissue acquisition via biopsy or resection for mitotic index evaluation [9, 10]. Of note the expanding use of neoadjuvant therapy though effective for tumor size reduction may significantly alter mitotic activity measurements potentially compromising stratification accuracy particularly in borderline cases.

Consequently, establishing a non-invasive, reproducible technique for preoperatively distinguishing GISTs from leiomyomas and optimizing treatment strategies for malignant GISTs carries important clinical implications.

Emerging developments in radiomics and artificial intelligence (AI) have transformed medical image interpretation through automated extraction of subvisual, high-dimensional imaging features, enabling noninvasive tumor characterization and prognostic prediction [11–14]. While these techniques have demonstrated clinical utility in thyroid, breast, and liver malignancies, their application to GIST, particularly for deep learning analysis of EUS images, remains an underdeveloped area warranting further investigation [15–19].

Accordingly, this study aimed to evaluate three distinct AI models incorporating different combinations of clinical factors, EUS imaging features, and radiomic signatures for GIST diagnosis, with particular focus on their differential performance in risk stratification and leiomyoma discrimination.

## Materials and methods

### Data source

This retrospective study was conducted in accordance with the Declaration of Helsinki and approved by the Ethics Committee of the First Affiliated Hospital of Chongqing Medical University (Approval No. 2024-384-01). None of the data included in this study has been used, shared, or published in any previous research, and there is no data overlap with other studies. The requirement for written informed consent was waived due to the retrospective nature of the study.

### Study population and design

**Fig. 1:**
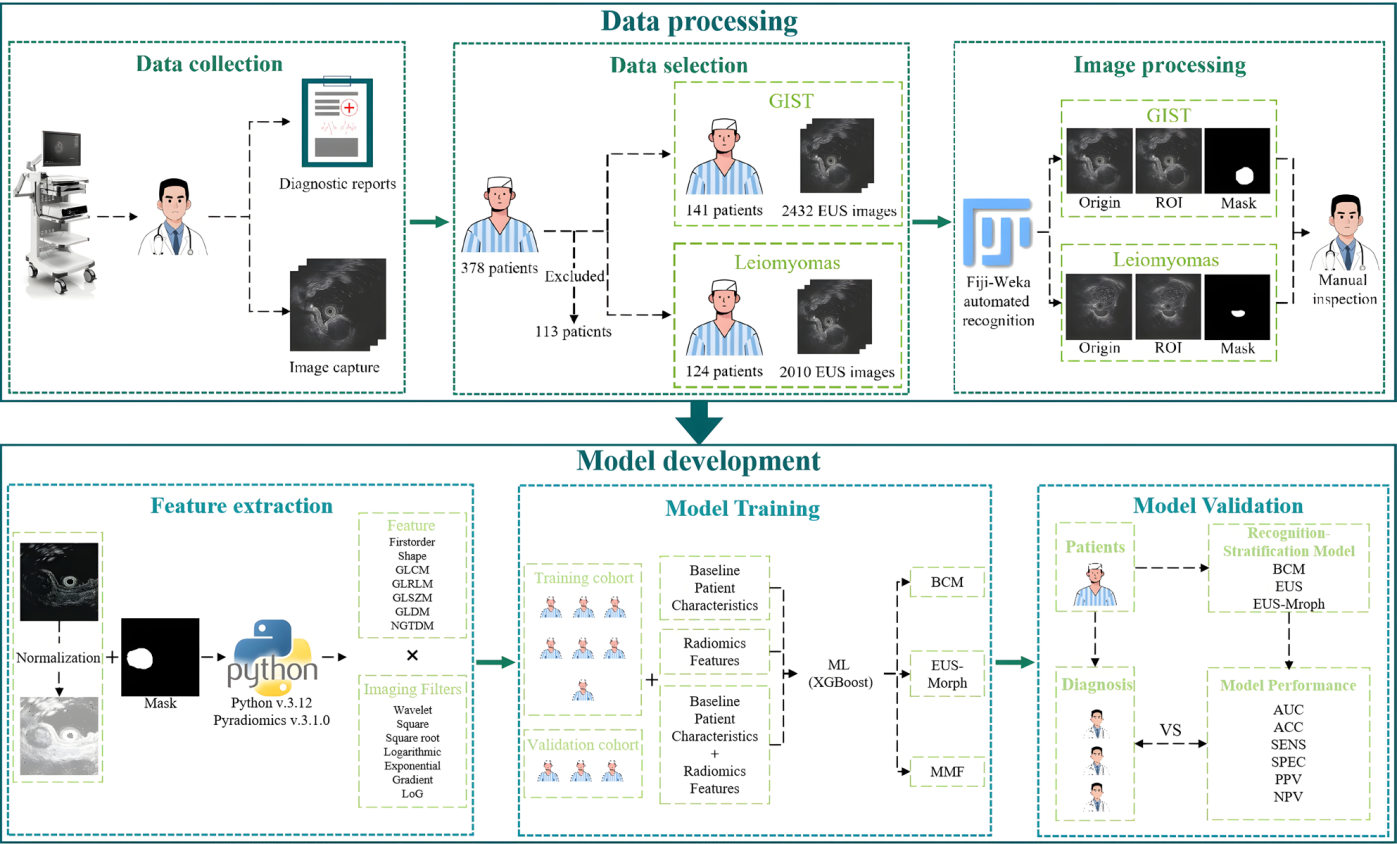
Overall study framework

**Fig. 2:**
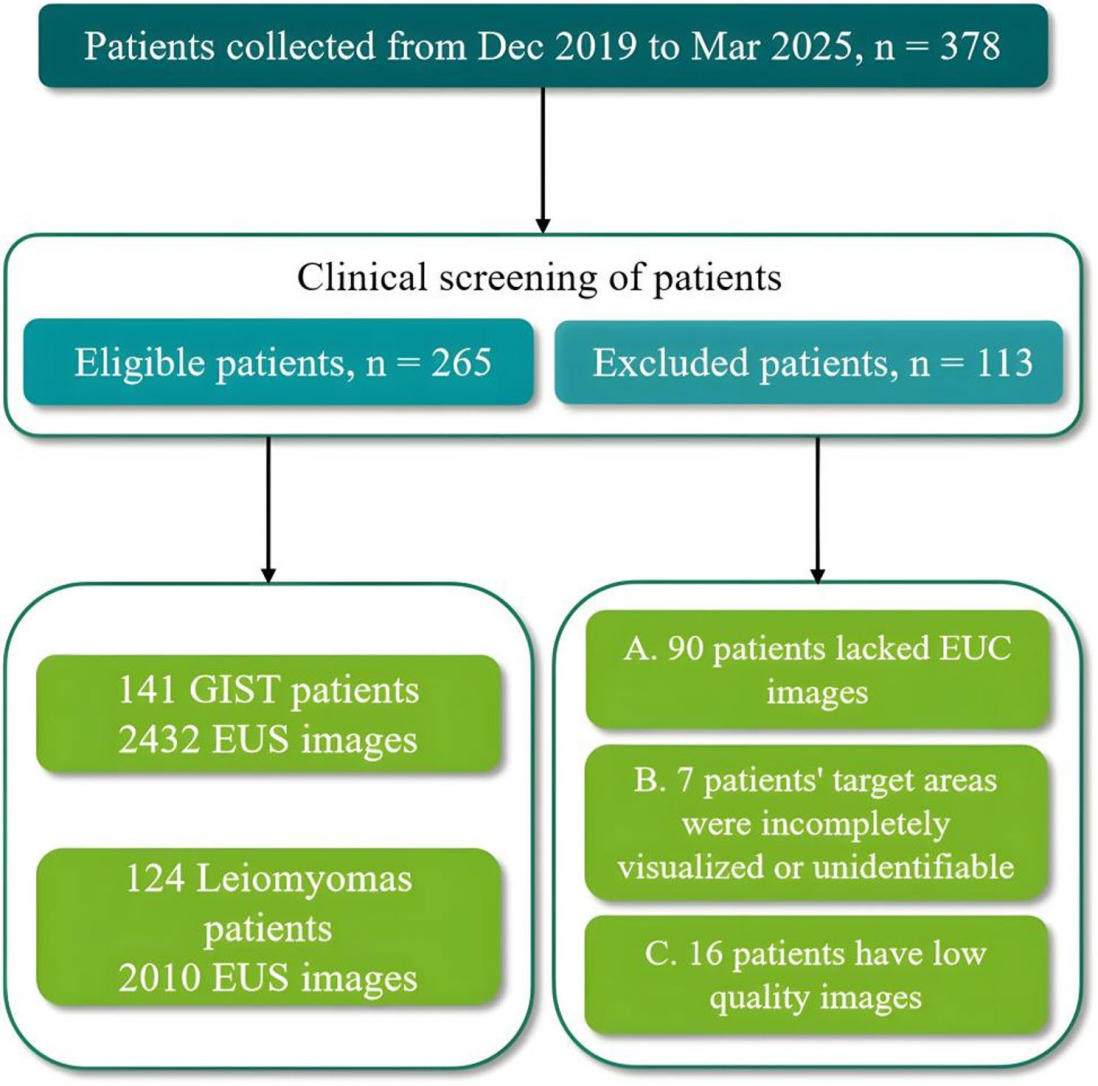
Flowchart of the patient selection process

This study consisted of four phases: patient enrollment, data processing, model development and validation, as illustrated in Fig. 1. From December 2019 to March 2025, 378 consecutive patients with histologically confirmed GIST or leiomyoma were identified from our institutional database for the derivation cohort. The pathological diagnosis was established by EUS-FNA or surgical resection. A structured retrospective analysis of the electronic medical records yielded preoperative parameters: patient age and sex, recorded clinical symptoms, and 2D-EUS-based morphological assessments. The inclusion criteria were: (1) complete pathological documentation (2) age > 18 years. Exclusion criteria included: (1) absence of preoperative EUS or missing images; (2) incomplete visualization of target lesions; (3) inadequate image quality for analysis. The final study cohort comprised 265 eligible patients. Of this group, 54.7% (145/265) were diagnosed by endoscopic ultrasound-guided fine-needle aspiration (EUS-FNA), while the remaining cases were confirmed through surgical resection. This cohort yielded a total of 4,442 endoscopic images (Fig. 2), comprising 2,432 images from 141 GIST patients and 2,010 from 124 patients with leiomyoma.

Patients were randomly allocated to training and validation sets in a 7:3 ratio. During model development, 10-fold cross-validation was implemented exclusively within the training cohort for both model training and feature selection. Final model performance was rigorously assessed on the completely independent validation set. Following identification of the optimal model, SHAP analysis was employed to elucidate its predictive decision-making process.

### Pathological gold standard and risk stratification

The study cohort included patients with initial diagnosis confirmed by either EUS-FNA or surgical resection. For those diagnosed via EUS-FNA, inclusion was contingent on subsequent curative surgical resection or complete endoscopic resection—procedures that provided sufficient tissue to enable reliable mitotic counting through assessment of 50 high-power fields. Consequently, the risk category predicted by our model was benchmarked against pathological findings from whole-tissue specimens, rather than relying solely on FNA-based cytology. All GIST diagnoses were validated using a standardized immunohistochemical panel incorporating CD117 (c-KIT), CD34, and DOG-1, with definitive diagnosis contingent upon positive immunoreactivity for CD117 and/or DOG-1.

Recurrence risk stratification was conducted using the modified NIH consensus criteria, which categorizes GIST cases into four distinct prognostic groups (very low, low, intermediate, and high) based on evaluation of tumor diameter, mitotic index, primary site, and rupture status. Following risk stratification, the training cohort was consolidated into two clinically relevant prognostic groupings: a low-risk group (encompassing both very low and low-risk categories) and a high-risk group (encompassing intermediate and high-risk categories) [10].

### Sonographic data acquisition

All EUS examinations in this retrospective study were performed using a standardized protocol with radial scanning echoendoscopes (Olympus EU-ME2 Premier platform) at 20 MHz, operated by certified endosonographers (≥ 2 years of experience). To minimize inter-operator variability, we implemented strict image inclusion criteria. Each patient was required to have at least one “diagnostically interpretable EUS image,” defined by clear visualization of the complete tumor border and the ability to accurately measure the long and short axes. All qualifying images were exported in DICOM format for analysis. The EUS imaging characteristics of GISTs and leiomyomas were systematically evaluated, including: tumor location, maximum diameter, long-to-short axis ratio (L/S ratio), layer of origin (muscularis propria, muscularis mucosa, or mixed), echogenicity (hypoechoic, iso- to hyperechoic, or mixed), homogeneity (homogeneous or heterogeneous), presence of hyperechoic foci, necrosis, and vascularity.

### EUS image processing pipeline

**Fig. 3:**
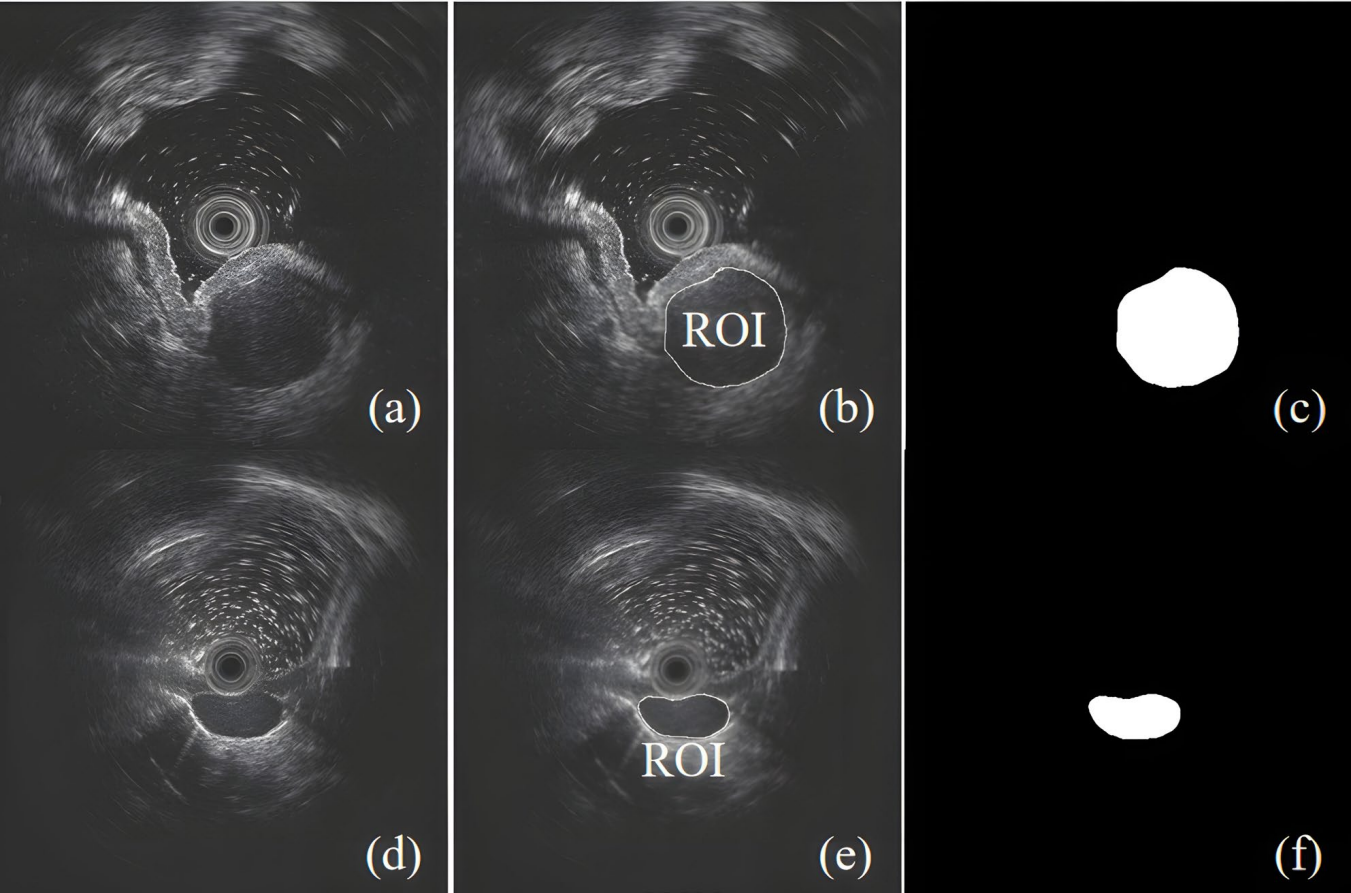
ROI segmentation process of EUS images for GIST(Top) and Leiomyoma (Bottom): (**a**)(**d**) Original EUS image; (**b**)(**e**) Segmentation of the tumor region; (**c**)(**f**) ROI mask

Tumor segmentation was performed by one senior endoscopist (> 5 years of EUS experience) and two junior endoscopists using Fiji ImageJ (v2.14) with the WEKA machine learning plugin, with initialization based on 50 histologically confirmed cases (25 GISTs, 25 leiomyomas). The senior specialist provided real-time guidance to ensure accuracy. Automated region-of-interest (ROI) generation was required to fully delineate tumor margins while excluding adjacent vasculature and fat infiltration. Discrepant contours were jointly reviewed and manually corrected by both operators until consensus was reached. Interobserver agreement was assessed via intraclass correlation coefficient (ICC; satisfactory threshold > 0.75). Final 8-bit grayscale masks were normalized (Python 3.12, scikit-image v0.22), achieving excellent spatial concordance (Dice > 0.9). Moreover, across the entire spectrum of radiomics workflows—encompassing image preprocessing procedures and region of interest (ROI) validation steps—the present study rigorously complied with the established guidelines formulated by the Image Biomarker Standardization Initiative (IBSI). This ensured the reproducibility of feature extraction and the comparability of results. Representative segmentations are displayed in Fig. 3.

### Feature extraction

Multiparametric radiomic signatures were yielded from EUS images using the feature extractor module in the open-source Pyradiomics package (version 3.1.0), based on tumor segmentation results. The yielded features included first-order intensity statistics, two-dimensional morphometric characteristics, and second-order texture parameters. Five grayscale matrices were utilized for structural texture feature analysis: the gray-level co-occurrence matrix (GLCM), gray-level dependence matrix (GLDM), neighborhood gray-tone difference matrix (NGTDM), gray-level run-length matrix (GLRLM), and gray-level size-zone matrix (GLSZM). To maximize image information extraction, seven imaging filters were additionally applied prior to feature extraction: Wavelet, Square, Square root, Logarithmic, Exponential, Gradient, and Laplacian of Gaussian (LoG) transforms. A total of 846 radiomic features were subsequently derived from each patient’s ultrasound image through this comprehensive processing pipeline. A random selection of 90 eligible cases was extracted from the study population for systematic evaluation of feature stability. ROI delineation was performed automatically by Fiji and manually by ultrasound specialists, followed by feature extraction. Feature stability was evaluated via intraclass correlation analysis, with only features demonstrating ICC ≥ 0.75 being incorporated for subsequent predictive modeling.

### Model development and validation

In this study, we developed diagnostic and risk stratification models for GIST using Python 3.12 and XGBoost 3.0.1. Three distinct models were constructed: (1) The Baseline Clinical Model (BCM) incorporating baseline clinical features and 2D-EUS morphological features (2) The Morphological Radiomics Model (EUS-Morph) based on EUS-derived radiomics features and (3) the Multimodal Fusion Model (MMF) integrating both datasets. To clinically contextualize the model’s performance, three independent board-certified endosonographers—blinded to all clinical information and AI outputs—independently assessed the same validation cohort. To minimize assessment bias, cases were presented in uniquely randomized sequences for each reviewer. Inter-observer agreement in both diagnosis and risk stratification was evaluated using weighted kappa statistics.

### Statistical analysis

All quantitative analyses were conducted using R statistical environment (v4.5.1; R Foundation) with complementary Python-based processing through Anaconda distribution (v2.6.6). Categorical variables were expressed as absolute counts with corresponding percentages (%), with intergroup differences evaluated by Pearson’s c^2^ tests. Continuous measures demonstrating normal distributions were reported as mean ± SD and compared using Student’s t-tests. Statistical inferences were considered significant when the two-sided probability value fell below the conventional 0.05 cutoff. Diagnostic performance was objectively assessed through receiver operating characteristic curve (ROC) analysis, with the area under the curve (AUC) representing the key performance metric. Secondary performance metrics comprised accuracy (ACC), sensitivity (SENS), specificity (SPEC), positive predictive value (PPV), and negative predictive value (NPV). A multifaceted validation paradigm was employed to rigorously assess the predictive model across key clinical dimensions: decision curve analysis (DCA) to assess clinical utility, precision-recall curve (PR) analysis to address class imbalance, and innovative radar plot visualization integrating five key performance metrics.

## Results

### Patient demographics and endosonographic characteristics

The baseline characteristics and EUS two-dimensional features are documented in Table 1. The analysis included 265 pathologically confirmed patients (141 GISTs vs. 124 leiomyomas). GIST patients were significantly older than leiomyoma patients (60.1 ± 9.8 vs. 53.2 ± 11.5 years, *p* < 0.001), while sex ratios were comparable (*p* > 0.999). Although maximum tumor diameter showed no significant difference (1.41 ± 1.04 cm vs. 1.17 ± 0.95 cm, *p* = 0.053), GISTs had significantly lower AP/TR ratio < 1 prevalence (70% vs. 87%, *p* = 0.001). Tumor origin analysis revealed GISTs predominantly originated from the muscularis propria (91% vs. 3%), whereas leiomyomas more frequently arose from the submucosa (27% vs. 1%, *p* < 0.001). Anatomically, GISTs were mainly located in the gastric fundus (55%) compared to esophageal predominance in leiomyomas (35%, *p* < 0.001). EUS characteristics showed GISTs had significantly lower hypoechogenicity (74% vs. 91%, *p* < 0.001), but higher mixed echogenicity (18% vs. 5.6%, *p* = 0.002), hyperechoic foci (15% vs. 4.8%, *p* = 0.002), and intratumoral vessels (8.5% vs. 0%, *p* = 0.002). GISTs exhibited worse homogeneity (83% vs. 98%, *p* < 0.001), while clinical symptoms (64% vs. 57%) and necrosis (4.3% vs. 0%) showed no statistically significant variation (*p* > 0.05). Variables demonstrating statistically significant associations (*p* < 0.05) were incorporated into the multivariable predictive model (see Table 1).

Patients were randomly assigned to training (*n* = 186) and validation (*n* = 79) cohorts in a 7:3 ratio. Baseline clinical characteristics and EUS imaging features showed no significant differences between GIST and leiomyoma subgroups in either cohort (all *p* > 0.05, Table 2), confirming appropriate cohort stratification.

### Radiomics and deep learning feature extraction

**Fig. 4:**
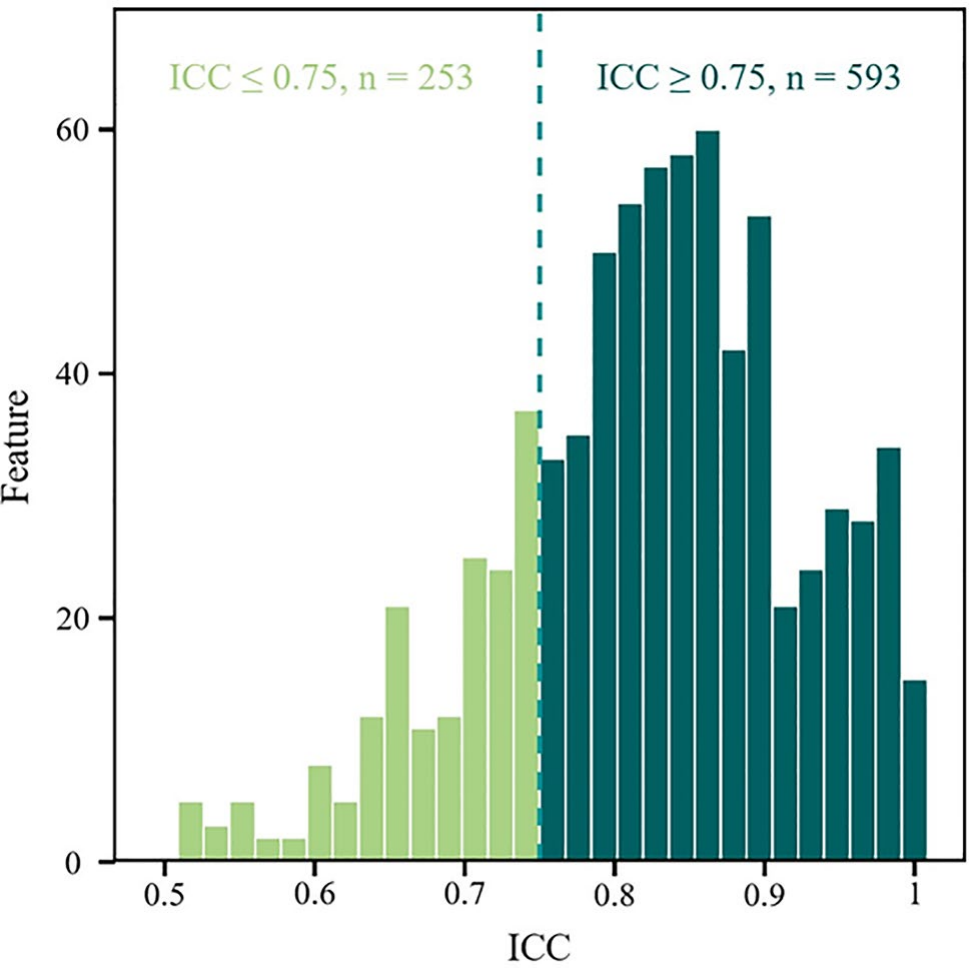
ICC of radiomic features

The analysis initially extracted 846 radiomics features, of which 593 demonstrated excellent inter-observer reliability (ICC ≥ 0.75) were retained, including 70 original features, 147 wavelet-transformed features, 40 square-transformed features, 69 square-root-transformed features, 74 logarithmically-transformed features, 69 exponentially-transformed features, 57 Gaussian-filtered features, and 67 gradient-based features, all of which were subsequently employed in model construction as illustrated in Fig. 4.

**Table 1:** Baseline characteristics and EUS features of patients with GIST and leiomyoma

Variable	Tumor Type		*P*-value
	GIST, *N* = 141	Leiomyoma, *N* = 124	
Age			< 0.001
Mean (SD)	60.1 (9.77)	53.1 (11.53)	
Maximum Tumor Diameter			0.053
Mean (SD)	1.4 (1.04)	1.2 (0.95)	
Sex			> 0.999
Female	98 (70%)	86 (69%)	
Male	43 (30%)	38 (31%)	
L/S ratio			0.001
<1	99 (70%)	108 (87%)	
=1	27 (19%)	6 (4.8%)	
>1	15 (11%)	10 (8.1%)	
Tumor Origin			< 0.001
Mixed-origin	0 (0%)	10 (8.1%)	
Muscularis propria	128 (91%)	81 (65%)	
Muscularis mucosa	13 (9.2%)	33 (27%)	
Tumor Localization			< 0.001
Esophagus	1 (0.7%)	44 (35%)	
Gastric antrum	6 (4.3%)	1 (0.8%)	
Gastric body	47 (33%)	46 (37%)	
Gastric cardia	3 (2.1%)	9 (7.3%)	
Gastric fundus	77 (55%)	19 (15%)	
Multifocal Lesion	5 (3.5%)	5 (4.0%)	
Other regions	2 (1.4%)	0 (0%)	
Homogeneity	117 (83%)	122 (98%)	< 0.001
Echogenicity			0.002
Iso- to hyperechoic	10 (7.1%)	4 (3.2%)	
Hypoechoic	105 (74%)	113 (91%)	
Mixed echogenicity	26 (18%)	7 (5.6%)	
Hyperechoic foci	21 (15%)	6 (4.8%)	0.013
Necrosis	6 (4.3%)	0 (0%)	0.056
Blood Flow	12 (8.5%)	0 (0%)	0.002
Associated Clinical Symptoms	90 (64%)	71 (57%)	0.333

**Table 2:** Comparison of baseline and EUS features between GIST and leiomyoma in training and validation cohorts

Characteristics[cases(%)]		Training cohor(*n* = 186)	Validation cohor(*n* = 79)	*P*-value
Age				0.2625
Mean (SD)		57.4(11.1)	55.7(11.1)	
Maximum Tumor Diameter				0.8639
Mean (SD)		1.3(1.01)	1.3(0.97)	
L/S ratio ≥ 1		41(22.0%)	17(21.9%)	1
Necrosis(Yes)		91(48.9%)	37(46.8%)	0.8900
Blood Flow(Yes)		93(50.0%)	42(53.2%)	0.7659
Hyperechoic foci(Yes)		105(56.5%)	40(50.6%)	0.4896
Sex	Female	130(69.9%)	54(68.4%)	0.8849
	Male	56(30.1%)	25(31.6%)	
Echogenicity	Iso- to hyperechoic	5(2.7%)	2(2.5%)	0.9351
	Mixed echogenicity	35(18.8%)	14(17.7%)	
	Hypoechoic	146(78.5%)	63(79.7%)	
Homogeneity	Yes	81(43.5%)	42(53.2%)	0.2088
	No	105(56.5%)	37(46.8%)	
Tumor Origin	Muscularis propria	145(78.0%)	64(81.0%)	0.4896
	Muscularis mucosa	35(18.8%)	11(13.9%)	
	Mixed-origin	6(3.2%)	4(5.1%)	
Tumor Localization	Gastric fundus	68(36.6%)	29(36.7%)	0.7748
	Gastric body	66(35.5%)	28(35.4%)	
	Gastric antrum	2(1.1%)	2(2.5%)	
	Gastric cardia	11(5.9%)	3(3.8%)	
	Esophagus	30(16.1%)	15(19.0%)	
	Other regions	9(4.8%)	2(2.5%)	
Associated Clinical Symptoms	Yes	76(40.9%)	27(34.2%)	0.3516
	No	110(59.1%)	52(65.8%)	

### Performance of the BCM、EUS-Morph and MMF models

Three XGBoost-based predictive models were developed using different feature sets: the BCM model, the EUS-Morph model, and the MMF model. These models aimed to distinguish between GIST and leiomyoma and to stratify the risk levels of GIST. Comparative performance metrics for the three models and endoscopists’ diagnostic accuracy and risk stratification are summarized in Tables 3 and 4.

**Fig. 5:**
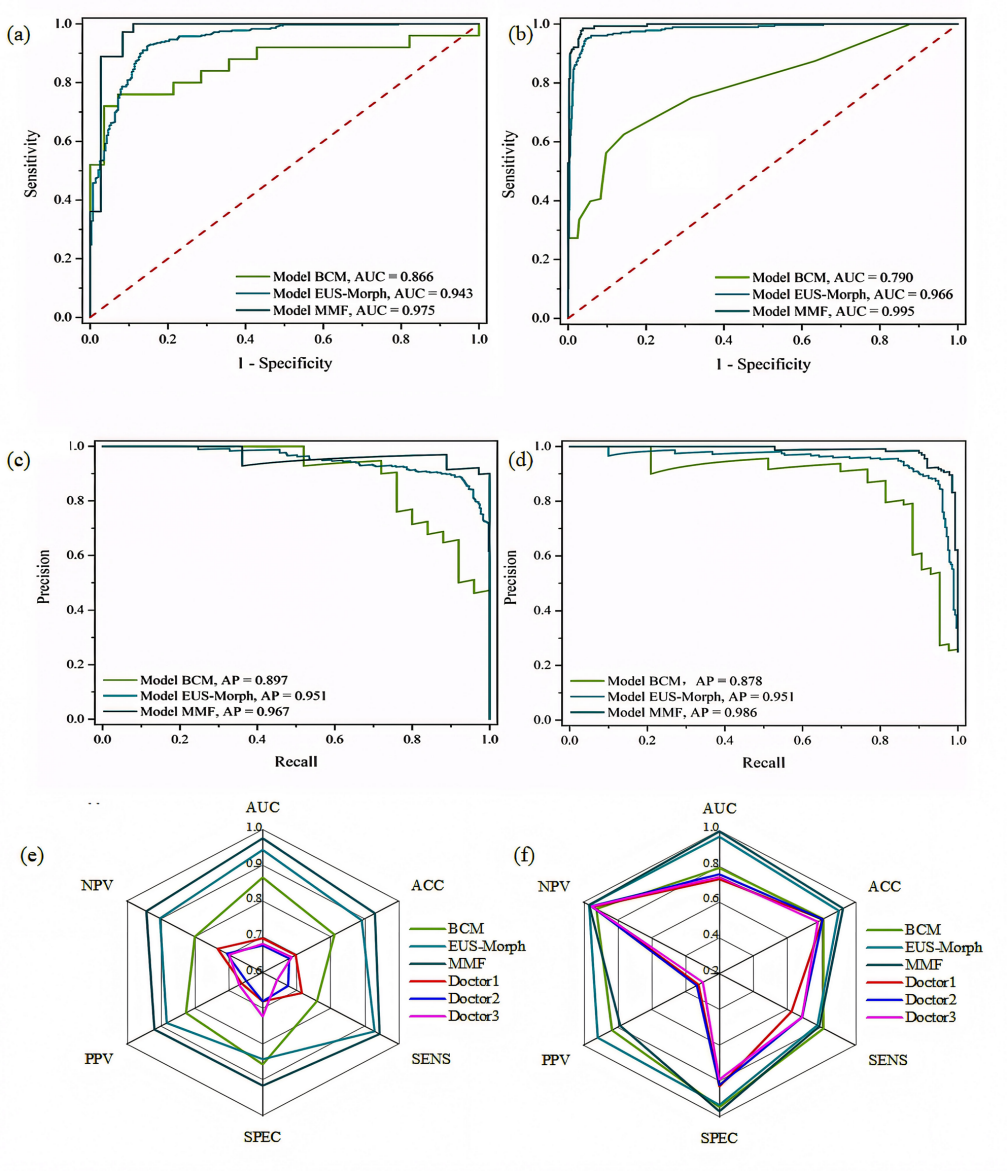
Multi-perspective evaluation of GIST diagnosis (left) and risk prediction (right). (**a**),(**b**): ROC curve, models whose curves deviate more significantly from the red baseline demonstrate superior performance; (**c**),(**d**):PR curves, If the PR curve of 1 model is completely covered by the PR curve of another model, it can be concluded that the latter is superior to the former; (**e**),(**f**) Radar plot, a larger enclosed area indicates better overall model performance

**Fig. 6:**
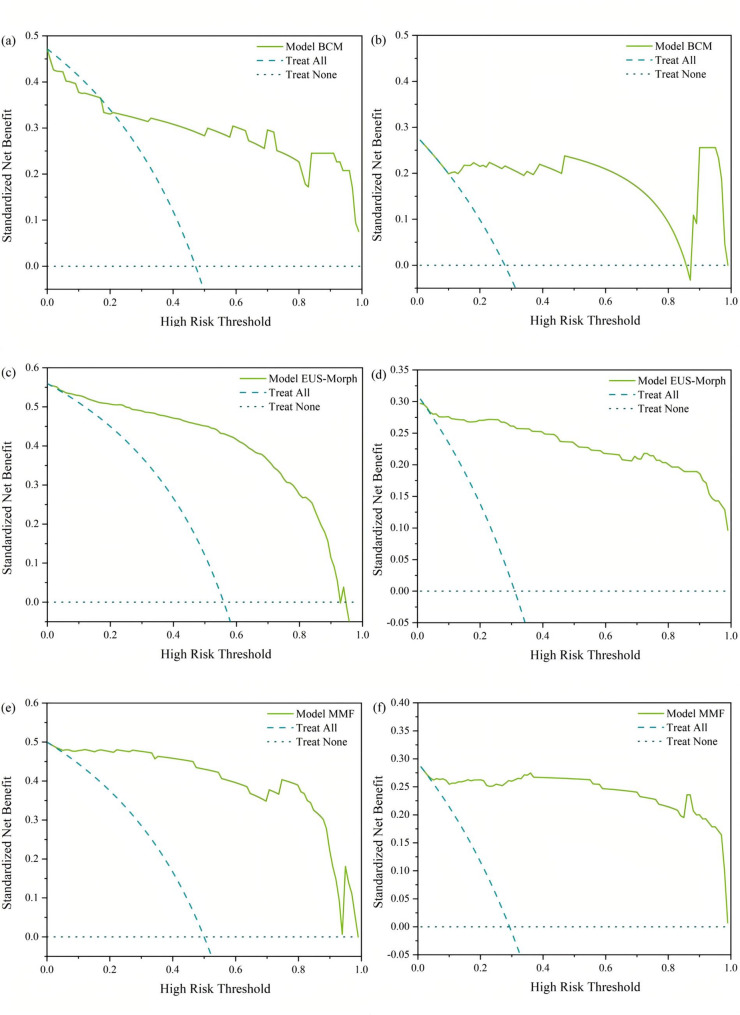
DCA comparing GIST diagnostic (left) and risk stratification models (right). The range where a model’s curve lies above both the “Treat All” and “Treat None” reference lines indicates clinically meaningful utility

The inter-rater reliability among endoscopists showed moderate concordance for GIST diagnosis (κ = 0.46), reflecting the documented challenges in EUS-based discrimination of these neoplasms. Conversely, NIH risk stratification attained substantial agreement (κ = 0.625), indicating clinically relevant consensus for this inherently subjective assessment and establishing a robust reference standard for objective comparison with computational models. Model evaluation results, including ROC curves, PR curves, multidimensional radar plot visualizations, and DCA, are presented in Figs. 5 and 6, respectively. All three models demonstrated robust performance, with the MMF model exhibiting better performance across all evaluation metrics. ROC analysis revealed outstanding diagnostic and risk-stratification capabilities, with AUC values of 0.975 (95% CI: 0.929–0.999) for diagnosis and 0.995 (95% CI: 0.988–0.999) for stratification, significantly surpassing other models (all *p* < 0.001). PR curve analysis confirmed consistent performance across recall levels (AP = 0.967), indicating reliable positive/negative differentiation despite imbalanced data distribution. Multidimensional radar plot visualization quantitatively confirmed balanced performance superiority. DCA demonstrated significantly higher net benefit across all threshold probabilities compared to treat-all and treat-none strategies (*p* < 0.01), validating its clinical applicability. This multi-modal validation confirms the dual advantages of the MMF model in GIST management, demonstrating both statistical robustness and clinical applicability. Comparative analysis with endoscopist performance (Tables 3 and 4) showed the MMF model achieved diagnostic metrics (AUC = 0.975, accuracy = 0.931, sensitivity = 0.944, specificity = 0.917, PPV = 0.919, NPV = 0.943) and stratification metrics (AUC = 0.995, accuracy = 0.929, sensitivity = 0.789, specificity = 0.971, PPV = 0.784, NPV = 0.967). In contrast, endoscopists showed diagnostic AUCs of 0.695, 0.675, and 0.680 and stratification AUCs of 0.728, 0.756, and 0.740 (all *p* < 0.001 vs. MMF).

**Table 3:** Superior diagnostic performance of the AI models over endoscopists for GIST identification

Diagnostic Modality	AUC (95% CI)	ACC	SENS	SPEC	PPV	NPV	Kappa
BCM	0.866 (0.752–0.961)	0.811	0.760	0.857	0.826	0.800	
EUS-Morph	0.943 (0.925–0.960)	0.892	0.930	0.843	0.883	0.902	
MMF	0.975 (0.929–0.999)	0.931	0.944	0.917	0.919	0.943	
Doctor 1	0.695 (0.632–0.759)	0.697	0.715	0.681	0.662	0.733	0.460
Doctor 2	0.675 (0.610–0.740)	0.678	0.675	0.681	0.648	0.706	
Doctor 3	0.680 (0.616–0.745)	0.686	0.642	0.723	0.670	0.699	

Note) BCM: Baseline Characteristics Model, EUS-Morph: Endoscopic Ultrasound-Morphology Model, MMF: Multimodal Model Fusion, AUC: area under the curve, ACC: accuracy, SENS: sensitivity, SPEC: specificity, PPV: positive predictive value, NPV: negative predictive value

**Table 4:** Enhanced GIST risk stratification by the AI models compared to endoscopists

Stratification Modality	AUC (95% CI)	ACC	SENS	SPEC	PPV	NPV	Kappa
BCM	0.791 (0.658–0.979)	0.814	0.814	0.947	0.833	0.925	
EUS-Morph	0.966 (0.941–0.985)	0.904	0.778	0.935	0.917	0.968	
MMF	0.995 (0.988–0.999)	0.929	0.789	0.971	0.784	0.967	
Doctor 1	0.728 (0.582–0.875)	0.809	0.625	0.832	0.323	0.946	0.625
Doctor 2	0.756 (0.613–0.898)	0.809	0.688	0.824	0.333	0.954	
Doctor 3	0.740 (0.595–0.885)	0.780	0.688	0.792	0.297	0.952	

Note) BCM: Baseline Characteristics Model, EUS-Morph: Endoscopic Ultrasound-Morphology Model, MMF: Multimodal Model Fusion, AUC: area under the curve, ACC: accuracy, SENS: sensitivity, SPEC: specificity, PPV: positive predictive value, NPV: negative predictive value

To elucidate the decision-making mechanism of the optimal multimodal fusion model, we employed the SHAP (SHapley Additive exPlanations) framework to enhance its interpretability (refer to Appendix 1, Figure S1). The SHAP summary plot identified blood flow features and Maximum Tumor Diameter as the most influential clinical factors for discriminating GIST and risk stratification, respectively. Among the radiomic features, original_glrim_RunEntropy and original_firstorder_RobustMeanAbsoluteDeviation were the most significant contributors to accurate diagnosis and classification. These features capture intratumoral structural heterogeneity by measuring textural heterogeneity and gray-level dispersion, respectively—imaging characteristics that are closely associated with malignant biological behavior and invasive potential. Furthermore, analysis of misclassifications revealed that errors predominantly occurred when SHAP contributions from different features were contradictory; for instance, cases exhibiting both high ‘Uniformity’ (a putative benign indicator) and high ‘Border Irregularity’ (a malignant indicator). This scenario mirrors diagnostic dilemmas encountered in clinical practice, underscoring the biological plausibility of the model’s decision-making process. The clinical and biological significance of key SHAP-derived features, with an emphasis on visually discernible characteristics, is systematically cataloged in Appendix 3 (Tables S1-S2). This is complemented by case-based analyses in Appendix 1, where Tables S1-S2 provide the model’s output probabilities and final predictions for ten representative cases, and Tables S3-S4 detail interpretations of both accurate and erroneous predictions across diagnostic and prognostic tasks.

## Discussion

Distinguishing and risk-stratifying GISTs carries significant clinical implications. Misclassification may delay targeted therapy and impact long-term survival, whereas overdiagnosis could lead to unnecessary surgical interventions. Although artificial intelligence and radiomics have advanced diagnostic precision across various diseases, their application in EUS-based GIST assessment remains limited [20–23]. Against this backdrop, we developed and validated three integrated EUS-based fusion models, whose value can be evaluated through three key dimensions. Methodologically, our study establishes an optimized modeling strategy through multimodal data integration. Existing approaches, including those by Zhang et al. [24] and Lu et al. [25], primarily rely on conventional morphological features for GIST identification, while Zhuo et al. [26] focused solely on ultrasound radiomics for recurrence prediction. In contrast, our framework systematically integrates baseline characteristics, clinical parameters, EUS morphological features, and radiomics signatures to construct a comprehensive diagnostic model. Quantitative analyses demonstrate that the multimodal radiomics approach outperforms conventional single-modality assessments [27]. Clinically, EUS-based radiomics offers distinct advantages in evaluating subepithelial lesions. The high-resolution capability of EUS enables detailed visualization of gastrointestinal wall structures, while radiomics provides quantitative characterization of lesion heterogeneity. This combined approach supports real-time diagnosis and risk stratification during endoscopic procedures, assisting operators in determining the need for tissue sampling or assessing resection feasibility. For validation, we implemented a clinically-oriented evaluation framework. Beyond conventional metrics, direct comparison with endoscopic experts and real-world testing established the model’s utility and complementary role in diagnosis and risk prediction Furthermore, unlike studies dependent solely on ROC analysis [28–31], we introduced a comprehensive framework incorporating precision-recall curves for class imbalance and decision curve analysis to quantify clinical benefit. Six-dimensional radar charts enabled intuitive multi-metric performance visualization. This integrated methodology provides robust translational evidence for clinical implementation of AI models.

Our study demonstrates that an integrated AI and radiomics model incorporating baseline clinical characteristics and EUS imaging features significantly improves both diagnostic and risk stratification performance for GISTs. Quantitative analyses revealed that the MMF model achieved at least a 40% higher diagnostic AUC (0.975 vs. 0.695) and at least a 32% higher risk stratification AUC (0.995 vs. 0.756), compared to endoscopists, with non-overlapping confidence intervals indicating clinically meaningful improvement.

Based on the design and implementation framework of this study, we have systematically examined and categorized the limitations into clinical and technical aspects. Building upon this analysis, we have further elaborated the resulting future research directions. The most notable limitations include the single-center retrospective design and relatively limited sample size, which constrain the model’s generalizability and preclude comprehensive subgroup analyses. This study design is susceptible to spectrum bias—as a tertiary referral center, our patient cohort may not fully represent the broader population seen in community practice. We explicitly acknowledge that the absence of external validation constitutes a critical shortfall in assessing its real-world applicability. To address this, we are planning to collaborate with two independent medical centers to conduct a prospective validation study. This study will specifically evaluate model performance across different EUS platforms (including those from Olympus and Pentax) and varying levels of operator expertise. Furthermore, although the current model provides detailed probability outputs and post hoc interpretability, its clinical translation faces two core challenges: the absence of decision thresholds validated against clinical outcomes and the lack of systematic integration of patient-specific preferences. To address these limitations, we plan to use large-scale clinical outcome data in upcoming multicenter prospective studies to determine optimal risk score cut-off values and to develop a patient-specific decision framework that can be seamlessly integrated into routine workflow. This is expected to transform the model into a highly applicable clinical decision-support tool. On the technical side, the model also confronts two major challenges. First, its performance is highly dependent on the development environment. Having been developed and validated using specific endoscopic ultrasound devices and acquisition protocols, its stability may be compromised when applied to images acquired with different equipment parameters across institutions. Second, the inherent operator dependence of endoscopic ultrasound image quality may affect the reproducibility of feature extraction, despite our implementation of standardized region-of-interest processing.

Of note, 54.7% of cases in this cohort were initially diagnosed via EUS-FNA, which accurately reflects its established role as a primary diagnostic modality in contemporary clinical practice. Critically, the risk classification predicted by our model is benchmarked against the complete pathological evaluation of subsequent surgical or endoscopic resection specimens, not just FNA biopsy results. Consequently, it must be emphasized that this risk stratification approach is primarily applicable to GIST patients who had clinical indications for and ultimately underwent curative resection, and it has been rigorously validated in this patient group. In this context, the model does not replace definitive surgical pathology but serves as an adjunctive preoperative decision-support tool. It aims to provide quantitative risk assessment before curative resection to help clinicians plan subsequent clinical management, such as evaluating resection feasibility.

In conclusion, we developed and validated three models for GIST diagnosis and risk stratification, all demonstrating satisfactory predictive performance. Notably, The MMF model demonstrated exceptional discriminative ability, with diagnostic and predictive AUCs reaching 0.975 (95% CI: 0.929–0.999) and 0.995 (95% CI: 0.988–0.999), respectively. The findings indicate that the MMF model demonstrates significant effectiveness in GIST diagnosis and risk classification, supporting its clinical application for personalized management: high-risk patients underwent intensified monitoring, while low-risk cases avoided unnecessary interventions.

## Conclusion

In summary, as a noninvasive and radiation-free diagnostic approach, the BCM, EUS-Morph, and particularly the MMF model provide clinically valuable supplementary information for GIST diagnosis and risk stratification. These AI-assisted tools demonstrate high accuracy, operational efficiency, and noninvasiveness, offering critical support for clinical decision-making and personalized treatment selection. Their integration into routine endoscopic practice could significantly improve GIST management efficiency while reducing healthcare burdens.

## Supplementary Information

Below is the link to the electronic supplementary material.

Supplementary Material 1

Supplementary Material 2

Supplementary Material 3

## Data Availability

The datasets used in this study are not publicly available due to privacy but are available from the corresponding author on reasonable request.

## Declarations

### Supplementary material note

This study was designed and reported in accordance with the CLEAR checklist (PMID: 37142815, IF: 4.5, Q1) and METRICS checklist (PMID: 38228979, IF: 4.5, Q1) for radiomics research. The complete checklists and modeling source code are available in the supplementary material (Appendix 2) to ensure the transparency and reproducibility of the study. Additionally, we have revised the manuscript with reference to the recently published Radiomics Quality Score (RQS) 2.0 tool to further align with the latest standards for radiomics studies.

## Abbreviations

ACC: Accuracy
AFIP: Armed Forces Institute of Pathology Criteria
AI: Artificial Intelligence
AP: Average Precision
AUC: Areas under the curve
BCM: Baseline characteristics model
CI: Confidence Interva
DCA: Ddecision curve analysis
ESGE: The European Society of Gastrointestinal Endoscopy
EUS-Morph: Endoscopic ultrasound-based morphological model
GIST: Gastrointestinal stromal tumors
GLCM: Gray-level co-occurrence matrix
GLDM: Gray-level dependence matrix
GLRLM: Gray-level run-length matrix
GLSZM: Gray-level size-zone matrix
ICC: Intraclass correlation coefficient
LoG: Laplacian of Gaussian
MMF: Multimodal fusion model
NCCN: The National Comprehensive Cancer Network
NGTDM: Neighborhood gray-tone difference matrix
NIH: National Institutes of Health Criteria
NPV: Negative predictive value
PPV: Positive predictive value
PR: Precision-recall curve
ROC: Receiver operating characteristic curve
ROI: Region-of-interest
SELs: Subepithelial lesions
SENS: Sensitivity
SHAP: SHapley Additive exPlanations
SPEC: Specificity
