# Supplementary material for: Development and validation of an ultrasound-based AI-radiomics model for diagnosing and risk-stratifying gastrointestional stromal tumors: a retrospective diagnostic study
Source: BMC Med Imaging. 2025 Nov 27;25:493. doi: 10.1186/s12880-025-02050-z (PMC12659386; doi:10.1186/s12880-025-02050-z)
Supplement: Supplementary file 1 — Supplementary Material 1 [file 12880_2025_2050_MOESM1_ESM.pdf]

Appendix 1

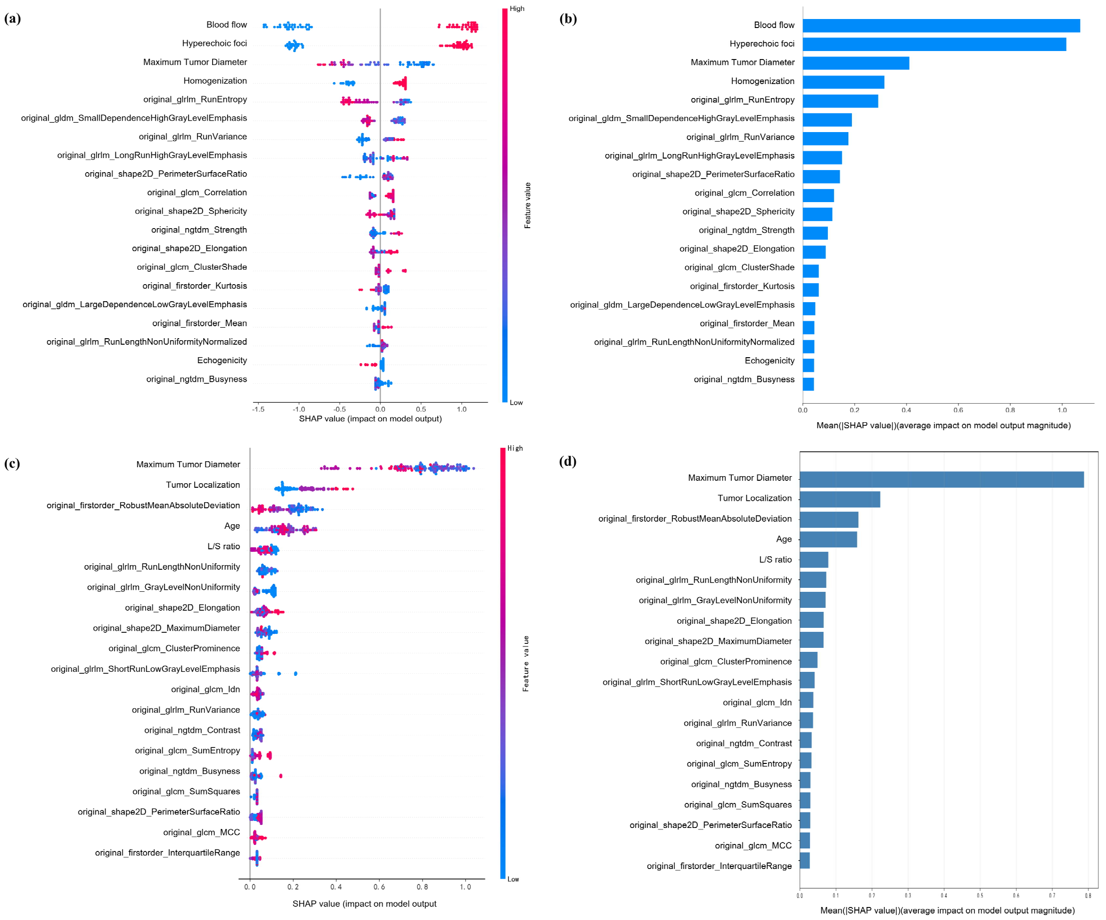

Figure S1. SHAP analysis for interpretability of the MMF model (see Appendix 1). (a) SHAP summary plot for the diagnostic task and (c) for the risk stratification task, each displaying the top 20 predictive features ranked by their global importance. Each point represents an individual sample, where its horizontal position indicates the SHAP value (direction and magnitude of feature impact), and color corresponds to the original feature value (red: high; blue: low). (b, d) Feature importance ranking plots for the diagnostic and stratification tasks, respectively. The bar length represents the mean absolute SHAP value for each variable, denoting its overall contribution to the model's output.

Table S1. Model Predictions on Representative Cases Based on SHAP Analysis

Model	Cases	Ture Label	Prediction Label	Prediction Probability	Prediction Correct
BCM	1	Leiomyoma	Leiomyoma	0.0275	Y
	2	GIST	GIST	0.9697	Y
	3	Leiomyoma	Leiomyoma	0.0086	Y
	4	GIST	GIST	0.9884	Y
	5	Leiomyoma	Leiomyoma	0.0896	Y
	6	GIST	Leiomyoma	0.4136	N
	7	Leiomyoma	Leiomyoma	0.0308	Y
	8	Leiomyoma	GIST	0.9096	N
	9	Leiomyoma	Leiomyoma	0.021	Y
	10	GIST	GIST	0.9945	Y
EUS-Morph	1	GIST	GIST	0.9710	Y
	2	Leiomyoma	Leiomyoma	0.0304	Y
	3	GIST	GIST	0.8489	Y
	4	Leiomyoma	Leiomyoma	0.0020	Y
	5	GIST	GIST	0.9954	Y
	6	Leiomyoma	Leiomyoma	0.0020	Y
	7	GIST	GIST	0.0637	Y
	8	GIST	Leiomyoma	0.4660	N
	9	Leiomyoma	Leiomyoma	0.0339	Y
	10	Leiomyoma	Leiomyoma	0.0445	Y
MMF	1	GIST	GIST	0.9877	Y
	2	GIST	GIST	0.8997	Y
	3	Leiomyoma	Leiomyoma	0.1082	Y
	4	Leiomyoma	Leiomyoma	0.0389	Y
	5	Leiomyoma	Leiomyoma	0.0170	Y
	6	GIST	GIST	0.8355	Y
	7	Leiomyoma	Leiomyoma	0.4526	Y
	8	Leiomyoma	Leiomyoma	0.1926	Y
	9	Leiomyoma	Leiomyoma	0.0726	Y
	10	Leiomyoma	Leiomyoma	0.0499	Y

Table S2. Verify the risk stratification effect of the model under SHAP

Model	Case ID	Ture Label	Prediction Label	Very Low Risk Probability	Low Risk Probability	Medium Risk Probability	High Risk Probability	Prediction Correct
BCM	22	Very Low Risk	Very Low Risk	0.8927	0.0571	0.0156	0.0345	Y
	9	Very Low Risk	Very Low Risk	0.7210	0.1266	0.0340	0.1183	Y
	23	High Risk	High Risk	0.0175	0.6356	0.0121	0.3348	Y
	3	High Risk	High Risk	0.0345	0.8242	0.0522	0.0891	Y
	32	Very Low Risk	Very Low Risk	0.6537	0.2063	0.0547	0.0853	Y
	24	Very Low Risk	Very Low Risk	0.8562	0.0763	0.0314	0.0361	Y
	20	Low Risk	Low Risk	0.0470	0.5960	0.3246	0.0324	Y
	1	Low Risk	Low Risk	0.0273	0.8612	0.0197	0.0917	Y
	16	Low Risk	Low Risk	0.0277	0.8951	0.0439	0.0332	Y
	18	Very Low Risk	Very Low Risk	0.6161	0.1330	0.1682	0.0827	Y
EUS-Morph	2	Very Low Risk	Very Low Risk	0.9856	0.0137	0.0003	0.0004	Y
	58	Very Low Risk	Very Low Risk	0.9952	0.0016	0.0027	0.0006	Y
	23	Very Low Risk	Very Low Risk	0.9956	0.0011	0.003	0.0003	Y
	18	Very Low Risk	Very Low Risk	0.9982	0.0008	0.0004	0.0006	Y
	17	Very Low Risk	Very Low Risk	0.9819	0.0023	0.0143	0.0015	Y
	41	Very Low Risk	Very Low Risk	0.9981	0.0008	0.0004	0.0007	Y
	12	Very Low Risk	Very Low Risk	0.9447	0.0094	0.0448	0.0012	Y
	85	Very Low Risk	Very Low Risk	0.9978	0.0017	0.0002	0.0003	Y
	27	Very Low Risk	Very Low Risk	0.9949	0.0017	0.0029	0.0005	Y
	62	Very Low Risk	Very Low Risk	0.9946	0.0019	0.0027	0.0008	Y
MMF	106	Very Low Risk	Very Low Risk	0.9379	0.0327	0.0113	0.0181	Y

91	Very Low Risk	Very Low Risk	0.9532	0.0163	0.0112	0.0193	Y
90	Low Risk	Low Risk	0.0437	0.8015	0.1192	0.0357	Y
50	Very Low Risk	Very Low Risk	0.9062	0.0435	0.0131	0.0372	Y
7	Low Risk	Low Risk	0.1101	0.8315	0.0278	0.0305	Y
103	Very Low Risk	Very Low Risk	0.8791	0.0788	0.0235	0.0185	Y
83	Very Low Risk	Very Low Risk	0.9249	0.0558	0.0084	0.0109	Y
104	Very Low Risk	Very Low Risk	0.9492	0.0169	0.0217	0.0122	Y
43	Very Low Risk	Very Low Risk	0.8937	0.0171	0.0148	0.0745	Y
59	Very Low Risk	Very Low Risk	0.9606	0.0131	0.009	0.0172	Y

16

Table S3. Diagnostic Classification of GIST versus Leiomyoma: Casewise Analysis with Model Interpretation

Case	Basic Clinical Information	Key Imaging Features	Model Prediction	SHAP Interpretation: Predictive Drivers
		**Correctly Classified Cases**		
**Case 1: GIST (High-Risk)**	• Age: 65<br>• Location: Gastric Body<br>•**Pathology:** GIST	• Diameter: 5.5 cm<br>• Hyperechoic Foci: Present<br>• Internal Echogenicity: Heterogeneous	**Prediction:**GIST	**Correct Attribution:** The prediction was primarily driven by heterogeneous echogenicity and presence of hyperechoic foci, aligning consistently with the pathological profile of high-risk GISTs
**Case 2: Leiomyoma (Benign)**	• Age: 48<br>• Location: Esophagus<br>•**Pathology:** Leiomyoma	• Diameter: 2.0 cm<br>• Hyperechoic Foci: Absent<br>• Internal Echogenicity: Homogeneous	**Prediction:**Leiomyoma	**Correct Attribution:** The prediction was dominated by lowRunEntropy (homogeneous texture) and smallMaximumDiameter(tumor size), consistent with features of a benign lesion.
		**Misclassified Cases**		
**Case 3: GIST (False Negative)**	• Age: 72<br>• Location: Gastric Antrum<br>•**Pathology:**GIST	• Diameter: 4.2 cm<br>• Hyperechoic Foci: Absent<br>• Internal Echogenicity: Homogeneous	**Prediction:**Leiomyoma	**Misattribution:** Despite the large size, the homogeneous texture (lowRunEntropy) exerted an excessively strong influence toward a benign prediction, overshadowing the risk signal from the large size resulting in a false negative.This reflects the diagnostic dilemma of homogeneous GISTs.
**Case 4: Leiomyoma (False Positive)**	• Age: 55<br>• Location: Gastric Fundus<br>•**Pathology:** Leiomyoma	• Diameter: 3.8 cm<br>• Hyperechoic Foci: Present<br>• Internal Echogenicity: Heterogeneous	**Prediction:**GIST	**Misattribution:** The internal heterogeneity (highRunEntropy) and presence of hyperechoic foci caused by degeneration were misinterpreted by the model as malignant features.This highlights the diagnostic challenge of benign lesions with secondary changes.

Table S4. Risk Stratification in GIST: Casewise Analysis with Model Interpretation

Case	Basic Clinical Information	Key Imaging Features	Model Prediction	SHAP Interpretation: Predictive Drivers
**Correctly Classified Cases**				
**Case 1:**<br>**GIST**<br>**(Low Risk)**	• Age: 66<br>• Location: Gastric Fundus<br>**•Diagnostic Risk:** Low Risk	•Diameter: 2.5 cm<br>• Hyperechoic Foci: Absent<br>• Internal Echogenicity: Homogeneous	**Prediction:**<br>Low Risk	**Correct Attribution:** The prediction was dominated by low heterogeneity (homogeneous echogenicity) and small tumor size (2.5 cm), consistent with typical features of low-risk GIST.
**Case 2:**<br>**GIST**<br>**(Very Low Risk)**	• Age: 45<br>• Location: Gastric Body<br>**•Diagnostic Risk:** Very Low Risk	• Diameter: 2.7 cm<br>• Hyperechoic Foci: Absent<br>• Internal Echogenicity: Heterogeneous	**Prediction**:<br>Very Low Risk	**Correct Attribution:** In GIST risk stratification, "small size (2.7 cm)" was the decisive factor. The critical negative finding of "absent hyperechoic foci" significantly mitigated the potential risk concerns raised by "heterogeneous echogenicity."
**Misclassified Cases**				
**Case 3:**<br>**GIST**<br>**(Low Risk)**	• Age: 53<br>• Location: Gastric Fundus<br>**•Diagnostic Risk:** Low Risk	• Diameter: 1.2 cm<br>• Hyperechoic Foci: Absent<br>• Internal Echogenicity: Homogeneous	**Prediction:**<br>Very Low Risk	**Misattribution:** The prediction model classified it as very low risk based on imaging features (small size, homogeneous). The final diagnosis, however, integrated the pathological gold standard (e.g., mitotic count), which provides a more precise basis for risk stratification.
**Case 4:**<br>**GIST**<br>**(Medium Risk)**	• Age: 54<br>• Location: Gastric Body<br>**•Diagnostic Risk:** Medium Risk	• Diameter: 0.6 cm<br>• Hyperechoic Foci: Absent<br>• Internal Echogenicity: Homogeneous	**Prediction:**<br>Low Risk	**Misattribution:** The final GIST risk category results from the combined assessment of "size" and "mitotic count." When these two indicators are discordant, the mitotic count carries greater weight than tumor size. Imaging can accurately measure size and describe structure but cannot replace the pathological assessment of cellular proliferative activity under the microscope.
