# Supplementary material for: Development and validation of an ultrasound-based AI-radiomics model for diagnosing and risk-stratifying gastrointestional stromal tumors: a retrospective diagnostic study
Source: BMC Med Imaging. 2025 Nov 27;25:493. doi: 10.1186/s12880-025-02050-z (PMC12659386; doi:10.1186/s12880-025-02050-z)
Supplement: Supplementary file 3 — Supplementary Material 3 [file 12880_2025_2050_MOESM3_ESM.pdf]

Table S1. Key Visual Features and Biological Rationale for GIST Diagnosis and Risk Stratification

Category	Core Visually Identifiable Features	Clinical and Biological Significance
**Diagnosis-Related**	Blood Flow	GISTs are typically hypervascular, a finding indicative of active angiogenesis that supports tumor growth.
	Homogenization	A homogenous internal echo pattern is a hallmark of benign leiomyomas, indicating their uniform cellular architecture, slow growth rate, and absence of necrosis or hemorrhage.
	Maximum Tumor Diameter	Tumor size is a well-established risk criterion, directly reflecting tumor burden and proliferative activity.
	Hyperechoic foci	Hyperechoic foci correspond to microscopic calcifications or fibrotic areas, signifying architectural remodeling and chronic inflammatory processes that attest to the lesion's intrinsic complexity.
	Echogenicity	While both GISTs and leiomyomas typically present as hypoechoic masses, the diagnostic key lies in the internal echo texture: heterogeneity suggests GIST, while homogeneity favors leiomyoma.
**Risk Stratification-Related**	Maximum Tumor Diameter	It directly quantifies tumor burden, serving as a surrogate for cumulative cell proliferation and genetic instability over time.
	L/S ratio	An elongated shape suggests an infiltrative growth pattern along tissue planes, indicative of locally aggressive behavior.
	Tumor Localization	Site-specific risk reflects the profound influence of the distinct tumor microenvironment on promoting aggressiveness.

Table S2. Categorization and Pathobiological Significance of Top 20 Diagnostic and Stratification Features Identified by SHAP Analysis

Category	Core Features	Clinical Significance	Biological Significance
**Tumor Morphological and Size Characteristics**	original_shape2D_MaximumDiameter	**Diagnosis:**Aids in differentiating GIST from leiomyoma. **Risk Stratification:** Larger tumor size indicates a higher risk level.	Reflects tumor proliferative activity; larger size correlates with greater mutational burden and invasive potential.
	original_shape2D_PerimeterSurfaceRatio	**Diagnosis:** Irregular borders help rule out leiomyoma. **Risk Stratification:** Increasing border irregularity correlates with higher risk.	Suggests infiltrative growth with tissue invasion, as opposed to a confined, encapsulated pattern.
	original_shape2D_Sphericity	**Diagnosis:** Non-spherical morphology is more suggestive of GIST.	Reflects disorganized, anarchic growth in GISTs versus the concentric, spatially constrained growth of leiomyomas.
	original_shape2D_Elongation	**Diagnosis:** An elongated shape is more predictive of GIST. **Risk Stratification:** Correlates with higher risk and infiltrative growth along tissue planes.	Indicates directional infiltration along muscle layers or tissue planes, rather than concentric expansion.
	L/S ratio	**Risk Stratification:** A markedly elongated morphology indicates higher risk, especially in confined sites (e.g., duodenum, rectum).	Reflects geometric constraints of the **anatomical** space, forcing axial growth that is concomitantly associated with enhanced invasiveness.
	Tumor Localization	**Risk Stratification:** Site-specific risk profiles exist (e.g., higher risk in duodenum/rectum vs. lower risk in esophagus), guiding follow-up and treatment.	Reflects differences in the tumor microenvironment; sites with rich vascularity and spatial constraints are more conducive to progression.
**Intratumoral**	Homogenization	**Diagnosis:**A heterogeneous echo pattern is highly suggestive of GIST, whereas homogeneity favors	Reflects internal cellular organization and composition. GISTs have disordered cells and necrosis causing heterogeneity;

Category	Core Features	Clinical Significance	Biological Significance
**Structural Heterogeneity Features**		leiomyoma.	leiomyomas have uniform architecture.
	Hyperechoic foci	**Diagnosis:**The presence of hyperechoic foci aids in ruling out leiomyoma, as they are more characteristic of GIST.	Corresponds to microscopic calcification or fibrotic areas, signifying architectural remodeling within the tumor.
	Echogenicity	**Diagnosis:**A hypoechoic appearance with internal heterogeneity is more suggestive of GIST.	Reflects cellular density and cytoplasmic complexity. GISTs are typically hypoechoic due to high cellularity, unlike the more homogeneous leiomyomas.
	original_glrlm_RunEntropy	**Diagnosis:**High entropy (signal disorder) is more predictive of GIST, helping quantify internal irregularity.	Quantifies the randomness of internal texture. High entropy indicates structural disorganization (e.g., mixed cells, necrosis) in GISTs.
	original_gldm_SmallDependenceHighGrayLevelEmphasis	**Diagnosis:**A high value is more suggestive of GIST, helping identify small, bright areas (e.g., micro-calcifications).	Reflects the distribution of small, high-intensity regions, which are more common in the complex structure of GISTs.
	original_glrlm_RunVariance	**Diagnosis:** High variance is more predictive of GIST.<br>**Risk Stratification:** High variance correlates with higher risk.	Reflects the heterogeneity in run lengths. High variance indicates co-existing dense cellular and necrotic areas, with more necrosis in high-risk GISTs.
	original_glrlm_LongRunHighGrayLevelEmphasis	**Diagnosis:**A high value is more suggestive of GIST, helping identify large, bright areas (e.g., extensive fibrosis).	Reflects the distribution of large, high-intensity regions, indicative of massive necrosis/fibrosis in GISTs.
	original_glcm_Correlation	**Diagnosis:**Low correlation is more predictive of GIST, helping quantify the disorderliness of tissue architecture.	Measures the linear dependency of neighboring pixels. Low correlation indicates disordered cell arrangement in GISTs.
	original_ngtdm_Strength	**Diagnosis:**High strength is more	Reflects the coarseness of the texture.

Category	Core Features	Clinical Significance	Biological Significance
		suggestive of GIST, helping identify the degree of local intensity differences.	High strength indicates major coarse patterns from structural heterogeneity in GISTs.
	original_glcm_ClusterShade	**Diagnosis:**High cluster shade is more predictive of GIST, helping identify the asymmetry of signal clusters.	Measures the skewness of the GLCM, indicating a lack of uniformity in the size of homogeneous regions in GISTs.
	original_firstorder_Kurtosis	**Diagnosis:**Low kurtosis is more suggestive of GIST, helping assess the peakedness of the intensity distribution.	Reflects the shape of the intensity distribution. Low kurtosis (flatter distribution) indicates multiple tissue components in GISTs.
	original_firstorder_Mean	**Diagnosis:**A lower mean intensity is more predictive of GIST, aiding in assessing overall tumor brightness.	Reflects the average echogenicity. GISTs are often darker due to high cellularity affecting sound transmission.
	original_glrlm_RunLengthNonUniformityNormalized	**Diagnosis:**High non-uniformity is more predictive of GIST, helping quantify the heterogeneity of run lengths.	Measures the heterogeneity of run lengths. High values indicate uneven distribution of homogeneous run lengths in GISTs.
	original_ngtdm_Busyness	**Diagnosis:** High busyness is more suggestive of GIST.<br>**Risk Stratification:**High busyness correlates with higher risk.	Reflects the frequency of local intensity changes. High busyness indicates rapidly changing textures from complex structures in aggressive GISTs.
	original_firstorder_RobustMeanAbsoluteDeviation	**Risk Stratification:**A high value correlates with higher GIST risk, helping assess internal structural stability.	Reflects the dispersion of the intensity values. A high value indicates numerous necrotic/hemorrhagic areas and structural instability.
	original_glrlm_RunLengthNonUniformity	**Risk Stratification:**High non-uniformity correlates with higher GIST risk, helping identify disparities in run length distribution.	Measures the heterogeneity of run lengths throughout the image. High values signify major differences between regions in high-risk GISTs.
	original_glrlm_GrayLevelNonUniformity	**Risk Stratification:**High	Measures the variability of gray-level

Category	Core Features	Clinical Significance	Biological Significance
		non-uniformity correlates with higher GIST risk, helping assess the heterogeneity of intensity levels.	intensities. High values indicate greater intratumoral heterogeneity in aggressive GISTs.
	original_glcm_ClusterProminence	**Risk Stratification:**High prominence correlates with higher GIST risk, helping identify the skewness and asymmetry of the GLCM.	Measures the skewness of the GLCM. High prominence indicates less symmetry and major structural asymmetry in high-risk GISTs.
	original_glrlm_ShortRunLowGrayLevelEmphasis	**Risk Stratification:**A high value correlates with higher GIST risk, helping identify small, dark areas (e.g., micro-necrosis).	Reflects the distribution of short, low-intensity runs. A high value suggests abundant micro-necrotic foci in aggressive GISTs.
	original_glcm_Idn	**Risk Stratification:**A low value correlates with higher GIST risk, helping quantify the local homogeneity of the image.	Measures local homogeneity. A low value indicates disordered local structures and greater heterogeneity in high-risk GISTs.
	original_ngtdm_Contrast	**Risk Stratification:**High contrast correlates with higher GIST risk, helping identify the disparity between neighboring pixels.	Reflects local intensity variations. High contrast indicates clear distinctions between necrotic and viable tumor areas in aggressive GISTs.
	original_glcm_SumEntropy	**Risk Stratification:**High entropy correlates with higher GIST risk, helping assess the overall randomness of the image texture.	Measures the randomness of the sum of pairwise pixel intensities. High entropy indicates complex and disordered structures in high-risk GISTs.
	original_glcm_SumSquares	**Risk Stratification:**A high value correlates with higher GIST risk, helping quantify the overall dispersion of the GLCM.	Reflects the variance of the GLCM. A high value indicates greater textural heterogeneity in aggressive GISTs.
	original_glcm_MCC	**Risk Stratification:**A low value correlates with higher GIST risk, helping assess the complexity of the	Measures the complexity of the texture. A low value indicates less linear dependency and more complex,

Category	Core Features	Clinical Significance	Biological Significance
		linear relationship in the image.	disordered patterns in high-risk GISTs.
	original_firstorder_InterquartileRange	**Risk Stratification:**A high value correlates with higher GIST risk, helping assess the spread of the middle intensity values.	Reflects the spread of the central intensity distribution. A high value indicates a wider range of tissue components in aggressive GISTs.
**Clinically Relevant Features**	Blood flow	**Risk Stratification:** Hypervascularity aids in the differential diagnosis, as it is more suggestive of GIST.	Reflects the tumor's demand for angiogenesis to support its rapid growth, unlike the hypovascular leiomyomas.
	Age	Advanced patient age correlates with higher GIST risk, guiding adjustments to follow-up or treatment intensity.	Serves as a proxy for cumulative mutational burden and declining cellular repair capacity, increasing malignant transformation risk.
